# Pulmonary Artery Perfusion with Anti-Tumor Necrosis Factor Alpha Antibody Reduces Cardiopulmonary Bypass-Induced Inflammatory Lung Injury in a Rabbit Model

**DOI:** 10.1371/journal.pone.0083236

**Published:** 2013-12-27

**Authors:** Yang Yu, Mingxin Gao, Haitao Li, Fan Zhang, Chengxiong Gu

**Affiliations:** Department of Cardiac Surgery, Beijing An Zhen Hospital, Capital Medical University, Beijing, China; University of Kentucky, United States of America

## Abstract

Inflammatory lung injury is one of the main complications associated with cardiopulmonary bypass (CPB). Tumor necrosis factor-α (TNF-α) is one of the key factors mediating the CPB-induced inflammatory reactions. Our previous studies have shown that endotracheal administration of anti-tumor necrosis factor-α antibody (TNF-α Ab) produces some beneficial effects on lung in a rabbit CPB model. In this study, we further examined the effects of pulmonary artery perfusion with TNF-α Ab (27 ng/kg) on lung tissue integrity and pulmonary inflammation during CPB and investigated the mechanism underlying the TNF-α Ab-mediated effects in a rabbit model of CPB. Our results from transmission electron microscopy showed that the perfusion with TNF-α Ab alleviated CPB-induced histopathological changes in lung tissue. The perfusion with TNF-α Ab also prevented CPB-induced pulmonary edema and improved oxygenation index. Parameters indicating pulmonary inflammation, including neutrophil count and plasma TNF-α and malondialdehyde (MDA) levels, were significantly reduced during CPB by pulmonary artery perfusion with TNF-α Ab, suggesting that the perfusion with TNF-α Ab reduces CPB-induced pulmonary inflammation. We further investigated the molecular mechanism underlying the protective effects of TNF-α Ab on lung. Our quantitative RT-PCR analysis revealed that pulmonary artery perfusion with TNF-α Ab significantly decreased TNF-α expression in lung tissue during CPB. The apoptotic index in lung tissue and the expression of proteins that play stimulatory roles in apoptosis pathways including the fas ligand (FasL) and Bax were markedly reduced during CPB by the perfusion with TNF-α Ab. In contrast, the expression of Bcl-2, which plays an inhibitory role in apoptosis pathways, was significantly increased during CPB by the perfusion with TNF-α Ab, indicating that the perfusion with TNF-α Ab significantly reduces CPB-induced apoptosis in lung. Thus, our study suggests that pulmonary artery perfusion with TNF-α Ab might be a promising approach for attenuating CPB-induced inflammatory lung injury.

## Introduction

Cardiopulmonary bypass (CPB) is widely performed during open-heart surgery, however, the procedure can cause complications such as lung injury [1]. CPB-induced inflammatory reaction is thought to be the main reason causing the lung injury, and tumor necrosis factor-α (TNF-α) has been found to stimulate the CPB-induced inflammatory reaction [2]. In addition, a series of CPB-induced pathophysiological changes also can trigger apoptosis pathways, resulting in excessive apoptosis in lung and ultimately leading to lung dysfunction [3]. Thus, reduction of TNF-α function and CPB-induced apoptosis in lung seems to be a promising strategy to alleviate the inflammatory lung injury during CPB. In our previous study, we found that administration of anti tumor necrosis factor-α antibody (TNF-α Ab) by endotracheal intubation to rabbits undergoing CPB reduces the inflammation and apoptotic index of lung [4]. In addition, we also showed that intra-tracheal administration of TNF-α Ab to patients before and after CPB improves lung compliance and oxygen index and reduces leukocyte accumulation, TNF-α release, and malondialdehyde (MDA) production [5]. To further improve the efficiency and efficacy of the protective effects of TNF-α Ab and to further elucidate the mechanism underlying the TNF-α Ab-mediated effects, we performed pulmonary artery perfusion with TNF-α Ab on rabbits undergoing CPB. We found that pulmonary artery perfusion with TNF-α Ab could effectively reduce CPB-induced inflammatory lung injury by significantly reducing TNF-α expression in lung and altering the expression of proteins involved in apoptosis pathways, including Bax, FasL, and Bcl-2. Our study might provide new strategies for attenuation of CPB-induced inflammatory lung injury.

## Materials and Methods

### Ethics Statement

All the procedures regarding animal maintenance and experiments are in strict accordance with the policy of the Institutional Animal Care and Use Committee (IACUC) of Department of Veterinary Medicine of the Capital Medical University of China. The IACUC has approved this study. All surgery was performed under sodium pentobarbital anesthesia, and all efforts were made to minimize suffering.

### Cell Culture and Reagents

Polyclonal rabbit anti human TNF-α (TNF-α Ab) was purchased from Abcam (Cambridge, MA, USA). The antibody is purified using protein A beads. Human umbilical vein endothelial cells (HUVEC) were cultured in F99 media.

### Validation of the Binding and Function of TNF-α Ab

Two milliliter blood from the left atrium of a rabbit undergoing CPB was centrifuged at 3000 rpm for 10 min. Approximately 120 pg of TNF-α Ab bound on Protein A beads was added in 100 µl of the supernatant, and BSA blocked Protein A beads were added in the same amount of supernatant as the control. The samples were incubated at 37°C for 30 min, and then centrifuged at 10,000 rpm for 1 min. The supernatant was then collected. TNF-α level in the supernatant was measured by Enzyme-linked immunosorbent assay (ELISA, Biovision, USA).

MTT assay was performed to validate the function of the antibody. HUVEC were seeded in 96 well tissue culture plate at the density of 5000 cells per well and cultured in F99 media at 37°C and 5% CO_2_. After the culture became 90% confluence, the cells were treated with 1200 pg/ml TNF-α Ab only, 20 ng/ml human recombinant TNF-α only, or TNF-α Ab+TNF-α. After the cells were incubated for 24 h, 20 µl MTT solution (5 mg/ml, 0.5% w/v) was added in each well, and the cells were incubated for 4 h. Culture media and MTT solution were then gently removed, and 150 µl Dimethyl sulfoxide was added in each well. The plate was shaken for 10 min. Absorbance values at 490 nm were read on a plate reader.

### Animals

Forty healthy New Zealand white rabbits (weight 2.0–2.5 kg, male or female) were purchased from Beijing Liu Li He Ke Xing animal breeding and care center (Beijing, China). The rabbits were housed in the standard environment for animal care and without water uptake restriction.

### Rabbit CPB Model and Pulmonary Artery Perfusion

The 40 rabbits were randomly divided into 4 groups with 10 rabbits in each group. The rabbits of group I underwent CPB and received pulmonary artery perfusion with the perfusion solution, low potassium dextran (LPD) lung preservation solution only (CPB+LPD). The rabbits of group II underwent CPB and received pulmonary artery perfusion with TNF-α Ab diluted in LPD (CPB+TNF-α Ab). The rabbits of group III underwent CPB without pulmonary artery perfusion (CPB only). The rabbits of group IV underwent open chest operation with heparinization only without CPB (No CPB). The rabbit CPB model was established by carotid artery occlusion and venous catheterization according to our previous description [4], [6]. The rabbits were anesthetized with intraperitoneal injection of 3.5% of sodium pentobarbital. The perfusion needle was inserted into the aorta from the proximal end as soon as the aorta was cross-clamped, and the heart was perfused with the St. Thomas’ II cardioplegic solution (15–20 ml/kg). After the aorta was cross-clamped for 30 min, the heart was perfused with half volume of the cardioplegic solution for the second time. The aorta and pulmonary artery were cross-clamped for 60 min. CPB lasted for 120 min. For the rabbits that received the pulmonary artery perfusion with TNF-α Ab (Group II), the proximal end of the pulmonary artery was cross-clamped simultaneously as the ascending aorta was cross-clamped. TNF-α Ab (Abcam, USA) at the concentration of 1200 pg/ml (diluted in LPD) was perfused into rabbit lung from the distal end of the pulmonary artery. The tube used for the lung artery perfusion solution was kept in ice water to keep the lung perfusion solution at 4°C. The perfusion with TNF-α Ab was performed twice. The first perfusion (15 ml/kg) was performed as soon as the aorta was cross-clamped. After the aorta was cross-clamped for 30 min, the second perfusion with TNF-α Ab was performed (7.5 ml/kg). The rabbits of group I received the same volume and same manner of pulmonary artery perfusion with LPD only.

### Evans Blue Leakage Assay

Evans blue was dissolved in sterile saline at 2%. One hour after CPB or the chest was kept open, the rabbits were injected with Evans blue intravenously at 20 mg/kb. After 1 hour of complete dye circulation, the rabbits were euthanized with an overdose of anesthetic, and the lung was perfused with 50 mL pre-warmed phosphate buffered saline (PBS) via the right ventricle to remove the Evans blue dye from the blood vessels. Then the lung was dissected, weighed, cut into small pieces, and immersed in 3 mL formamide in a 50°C thermostatic water bath for 24 hour. After centrifugation for 15 minutes at 12,000 rpm, the supernatant was collected. The amount of Evans blue dye extracted in the formamide was determined by a spectrophotometry (UV spectrophotometer type 752, Shanghai first Optical instrument) at the wavelength of 620 nm. Distilled water was used as the blank. The optical density (OD) value was transformed into the concentration according to a standard curve of Evans blue dye. The Evans blue permeability rate (µg/g lung tissue) was calculated according to this equation: Evans blue concentration (µg/mL)×volume of formamide (ml) ÷ the total lung weight (g).

### Lung Tissue Specimen Preparation

Rabbit lung tissues of group I-III were collected before CPB, after CPB lasted for 30 min, and at CPB termination. Rabbit lung tissues of group IV were collected right after the chest was opened, after the chest was left open for 30 min, and for 120 min. Partial lung tissue of each sample was snap frozen in liquid nitrogen for future use, and the remaining tissue was measured the wet weight on an electronic balance and then was placed in 80°C oven for 48 h to thoroughly dry the tissues. The dry weight of the lung tissue was measured. Lung water content (%) was then calculated as the following equation: (wet weight – dry weight)/wet weight×100.

### Electron Microscopy

Lung tissue was cut into 1 mm^3^ pieces and fixed in 2.5% glutaraldehyde at 4°C for 4 hours. After washed with PBS for 3 times, the tissue pieces were fixed in 1% osmium tetroxide for 3 hours, and then washed again with PBS for 3 times, dehydrated, and immerged in the embedding resin, a mixture of dimethyl ketone and Epon812 (1∶2). The tissue pieces were sliced into ultra thin sections by using the Reichert-Jung Ultracut E Ultramicrotome (Leica Microsystems, Germany). The thickness of the tissue section was 50–100 nm. After dried, the tissue sections were then stained with uranyl acetate for 15 minutes, washed with distilled water thoroughly, stained with lead citrate for 15 minutes, and washed again with distilled water for 3 times. After dried, the tissue sections were examined under the transmission electron microscope, JEM-1200EX II TEM (JEOL, Japan).

### Blood Sample Collection

Blood samples were collected from both right and left atrium of the rabbits of groups I-III before CPB, 5 min after the release of the cross-clamp, and at CPB termination. For rabbits of group IV (No CPB), blood samples were collected from both right and left atrium when the chest was just opened, after the chest was left open for 60 min, and for 120 min.

### Determination of Oxygenation Index, Neutrophil Count, TNF-α, and MDA Levels in Blood Samples

The oxygenation index was calculated as the ratio of the arterial oxygen tension (PaO2)/fractional inspired oxygen (FiO2). Neutrophil count was determined by conventional microscopy. TNF-α and malondialdehyde (MDA) levels in blood samples were measured by Enzyme-linked immunosorbent assay (ELISA, Biovision, USA). The measured values were normalized to the hemoglobin (Hb) value using the following equation: normalized value = (Hb prior to CPB/Hb during CPB)×measurement value.

### Real Time Quantitative RT-PCR

Real time quantitative reverse transcription polymerase chain reaction (RT-PCR) was performed to analyze the mRNA level of TNF-α in lung tissue. Total RNA was isolated by the TRIZOL method. The concentration and quality of total RNA were determined by UV spectrophotometer. cDNA was synthesized from 1 µg of the total RNA by using the first strand cDNA synthesize kit (Promega, USA). The PCR primer sequence for TNF-α are 5′-AGGAAGAGTCCCCAAACA-3′ for the forward primer and 5′-GCAATGATCCCAAAGTAGA-3′ for the reverse primer. β-actin was used as the reference gene. The PCR primer sequence for β-actin are 5′-ACACGGTGCCCATCTACG-3′ for the forward primer and 5′-CAGGAAGGAGGGCTGGAA-3′ for the reverse primer. The PCR was performed at the following condition: initial denaturation at 95°C for 5 min, denaturation at 95°C for 30 sec, annealing at 55°C fro 30 sec, extension at 72°C for 60 sec, 35 cycles on the ABI 7500 FAST quantitative PCR instrument (Invitrogen, USA). Cycle threshold of TNF-α was first normalized to the reference gene, and then the level of TNF-α relative to that of the control (group IV no CPB when chest was just opened) was calculated using the equation: 2^ΔΔCT^. n = 10. The data were presented as mean ± SD.

### Apoptotic Index Determination

Apoptotic index of lung tissue was determined by the terminal transferase-mediated dUTP nick end labeling (TUNEL) staining using the DeadEnd™ Fluorometric TUNEL System (Promega, USA). The staining was performed according to the manufacturer’s manual for the kit. In brief, 10 µm frozen section of lung tissue was fixed in 4% paraformaldehyde (PFA) in PBS for 20 min, and then washed with PBS for 3 times. Lung tissue section was permeabilized with 0.2% Triton X-100 for 5 min and then washed with PBS for 3 times. The tissue section was stained with TUNEL-mix for 60 min at 37°C and washed. After washed with PBS, the tissue section was counterstained with DAPI for 5 min. The slide was mounted with fluorescence mounting medium. The tissue section was observed by confocal microscopy. Apoptotic index was calculated as the number of apoptotic cells per 1000 cells.

### Immunohistochemical Staining

The protein expressions of Bcl-2, Bax, and FasL were determined by immunohistochemical staining. Tissue section was fixed in 4% PFA and blocked in blocking serum. The tissue section was then incubated with primary antibody overnight, then washed and incubated with secondary antibody. Primary antibodies used in the staining included anti rabbit Bax, Bcl2, and FasL polyclonal antibody (Boshide Biotechnology LLC, Wuhan, China). Tissue section stained with secondary antibody only was used as the negative control. The stained tissue section was observed under microscope at the magnification of 400. Cells showing brown color in cytoplasm were considered positive for the staining. Five visual fields were randomly selected from each lung tissue section. Totally 200 alveolar epithelial cells were observed. Positive staining rate was calculated as the percentage of cells with positive staining over the total 200 cells. Tissue sections from each rabbit were observed and the average number of all the rabbits (n = 10) was calculated.

### Statistical Analysis

All the data were analyzed by the statistical analyzing software SPSS 17.0. The data were presented as mean ± standard deviation (SD). Comparison between groups was analyzed by one-way ANOVA and the least significant difference test. P<0.05 was considered significantly different.

## Results

**Figure 1:**
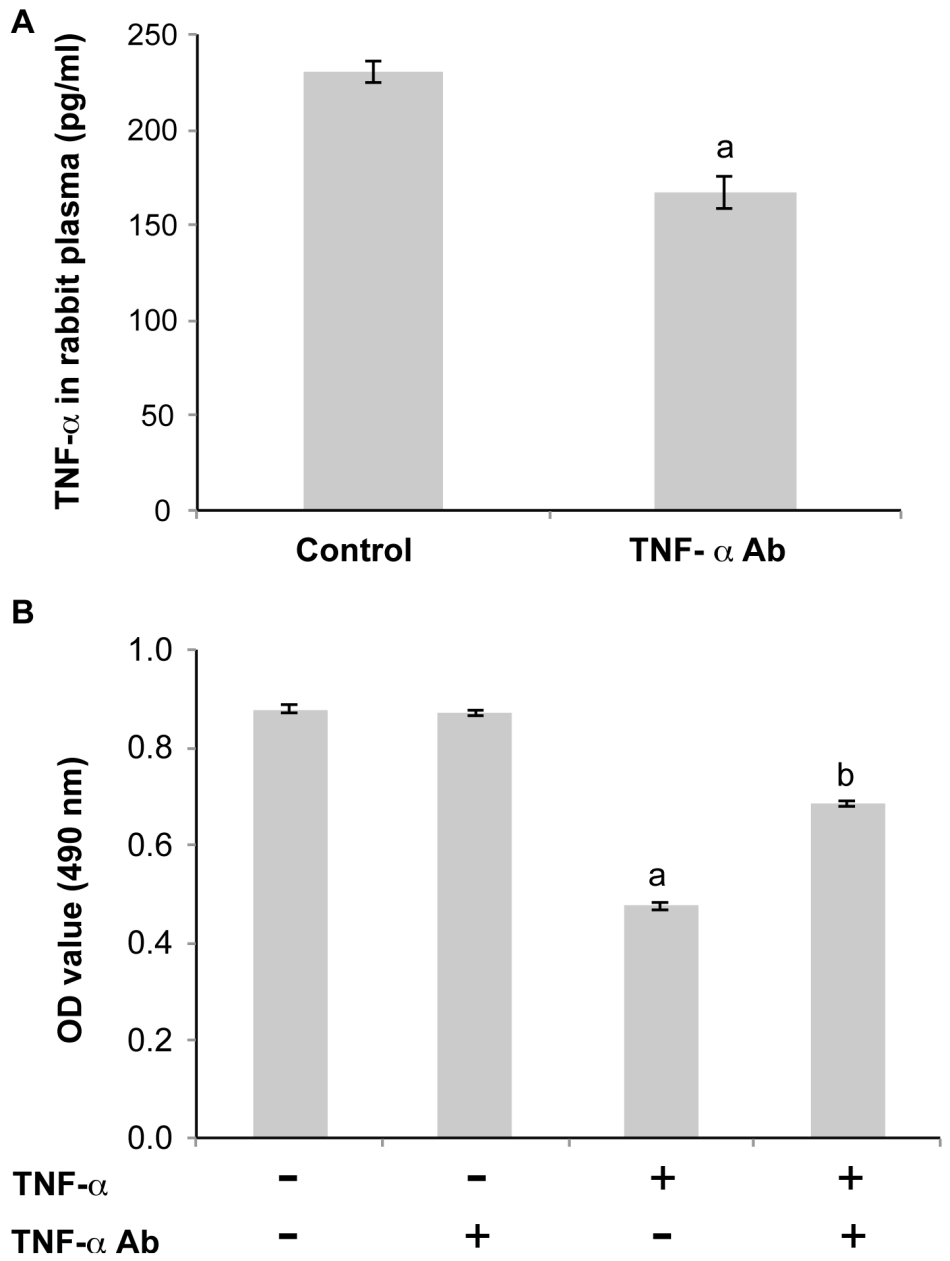
Validation of binding of TNF-α Ab to rabbit TNF-α and the functional blocking activity of the antibody. **A**. Incubation with TNF-α Ab significantly reduced TNF-α level in rabbit plasma. TNF-α Ab (120 pg) bound on Protein A beads or BSA blocked beads was incubated with 100 µl of the supernatant of rabbit plasma for 30 min at 37°C. TNF-α level was then measured by ELISA. ^a^P<0.05, students’ t-test. **B**. TNF-α Ab significantly decreased TNF-α-induced apoptosis in HUVEC. HUVEC on 96 well plate were treated with 1200 pg/ml TNF-α Ab only, 20 ng/ml human recombinant TNF-α only, or TNF-α Ab+TNF-α for 24 h. MTT assay was then performed to estimate HUVEC apoptosis. n = 3. Data were presented as mean ± SE.^ a^P<0.05, TNF-α only vs. control and TNF-α Ab only. ^b^P<0.05, TNF-α Ab+TNF-α vs. TNF-α only.

All the rabbits survived the CPB or the open chest operation. Twenty-seven of the thirty rabbits that underwent CPB had successful auto resuscitation after removal of the aortic cross-clamp, resulting in an auto resuscitation rate of 90.0% (27/30). For the three unsuccessfully auto resuscitated rabbits, two cases were due to incomplete cardiac arrest and insufficient myocardial protection, and one case had intraoperative bleeding and its auto resuscitation was adversely affected due to repetitive stretching of the heart to stop the bleeding. We first validated the binding of TNF-α Ab to rabbit TNF-α and the functional blocking activity of the antibody in vitro. Our result showed that TNF-α level in rabbit plasma was significantly reduced after incubation with TNF-α Ab (Figure 1A), suggesting that the antibody can bind rabbit TNF-α. We also performed MTT assay to estimate the effect of TNF-α Ab on TNF-α induced apoptosis in human umbilical vein endothelial cells (HUVEC). We found that TNF-α significantly inhibited the proliferation of HUVEC, while TNF-α Ab partially restore HUVEC proliferation, indicating that TNF-α Ab can block the TNF-α induced apoptosis in HUVEC (Figure 1B).

### Pulmonary Artery Perfusion with TNF-α Ab Alleviated CPB-induced Histopathological Changes in Lung Tissue

**Figure 2:**
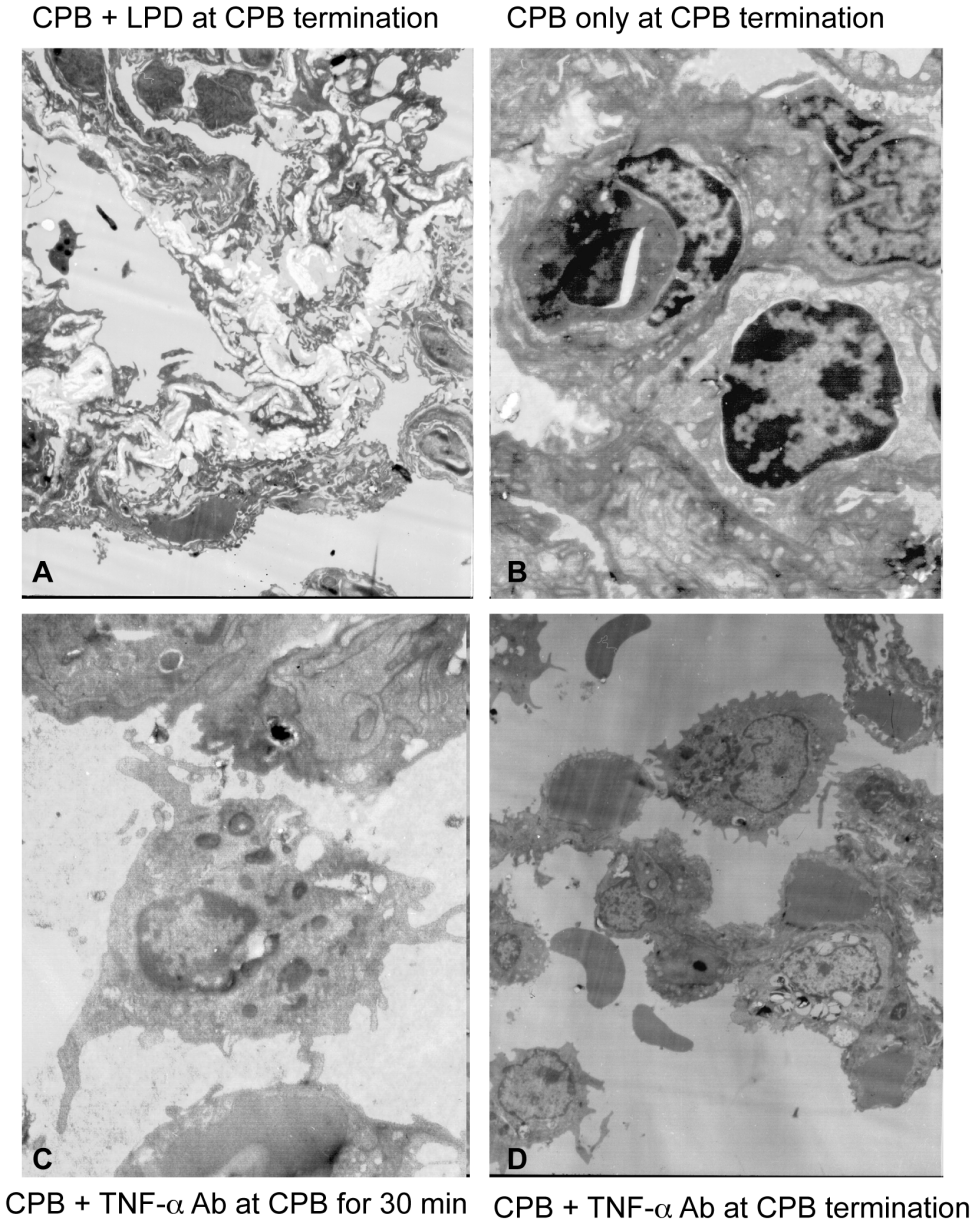
Pulmonary artery perfusion with TNF-α Ab alleviated CPB-induced histopathological changes in lung tissue. Lung tissue specimens from rabbits of group I (**A**), group II (**C** & **D**), and group III (**B**) were examined by transmission electron microscopy (JEM-1200EX II TEM, JEOL, Japan). The illustrated images (4000x) are the representative images.

**Figure 3:**
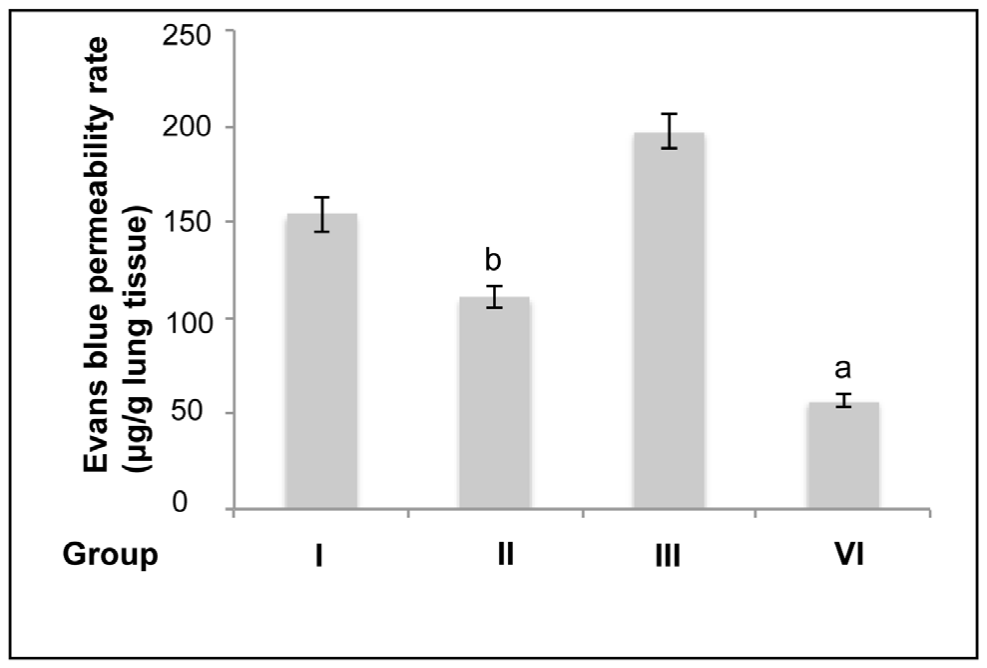
Pulmonary artery perfusion with TNF-α Ab reduced CPB-induced Evens blue leakage in lung. Group I: CPB+LPD; Group II: CPB+TNF-α Ab; Group III: CPB only; Group IV: Open chest no CPB. Evans blue (2%) was injected intravenously in rabbits one hour after CPB or the chest was kept open. Evans blue dye leaked into the lungs was extracted according to the description in the Methods. The Evans blue permeability rate (µg/g lung tissue) was calculated according to this equation: Evans blue concentration (µg/mL)×volume of formamide (ml) ÷ the total lung weight (g). n = 3. Data were presented as mean ± SE. ^a^P<0.05, group VI vs. group I, II, and III. ^b^P<0.05, group II vs. group I and III.

To assess the protective effects of pulmonary artery perfusion with TNF-α Ab on lung tissue during CPB, we first performed transmission electron microscopy to examine the ultrastructure of lung tissue. Figure 2A illustrated that large amounts of collagen deposition appeared in the alveolar septa of the rabbits that underwent CPB but without the perfusion with TNF-α Ab (Figure 2A). In addition, the number of type II alveolar epithelial cells was significantly reduced. The structure of blood-air barrier became disorganized and the basement membrane was partially ruptured in the rabbit undergoing CPB without the perfusion with TNF-α Ab (Figure 2A). Closer observation of the subcellular structure showed that the alveolar epithelial cells of the rabbits undergoing CPB only exhibited pyknosis and contained wide perinuclear space (Figure 2B). Furthermore, chromatin accumulated underneath the nuclear membrane. The inner mitochondrial membrane was degenerated, indicating the occurrence of apoptosis. Lamellar bodies and surfactant were reduced and alveolar collapsed. Tight cell-to-cell junction also disappeared. The type II alveolar epithelial cell layer became thinner in the rabbits undergoing CPB only (Figure 2B). In contrast, in the rabbits that underwent CPB and received pulmonary artery perfusion with TNF-α Ab, the type II alveolar epithelial cells exhibited leaf-shaped membrane protrusions, mild pyknosis, uniform euchromatin distribution, some vacuolization in cytoplasm, and intact organelles in cytoplasm (Figure 2C). The cytoplasm also contained rich mitochondria and lamellar bodies (Figure 2C). In addition, the number of type II alveolar epithelial cells was significantly increased compared to that in the rabbits undergoing CPB without the perfusion with TNF-α Ab. The structure of blood-air barrier was clear and the tissue did not exhibit significant apoptosis. The structure of alveoli was intact in the rabbits that underwent CPB and received the perfusion with TNF-α Ab (Figure 2D). These results suggest that pulmonary artery perfusion with TNF-α Ab could have beneficial effects on maintaining lung tissue integrity during CPB. We then performed the Evens blue leaking assay to evaluate CPB-induced lung damage and the protective effects of TNF-α Ab. Evans blue leakage was significantly increased by CPB (group I, II, and III vs. group IV, P<0.05), while pulmonary artery perfusion with TNF-α Ab reduced the CPB-induced Evens blue leakage significantly (group II vs. group I and III, P<0.05, Figure 3).

We then measured the lung water content. As highlighted in Table 1, CPB significantly increased the lung water content in the rabbit undergoing CPB but without the perfusion with TNF-α Ab compared to that in the rabbits without CPB (group I and III vs. group IV, P<0.05, Table 1). In contrast, the lung water content in the rabbits that underwent CPB and received pulmonary artery perfusion with TNF-α Ab was not significantly elevated and remained at the similar level as that in the rabbit without CPB (group II vs. group IV, P>0.05, Table 1). This result indicates that pulmonary artery perfusion with TNF-α Ab during CPB might prevent CPB-induced pulmonary edema.

**Table 1:** Lung water content measurement (mean ± SD, n = 10).

Group	I (CPB+LPD)	II (CPB+TNF-α Ab)	III (CPBonly)	IV (NoCPB)
Before CPB	77.93±1.27	78.35±2.04	77.76±2.12	78.78±3.47
CPBtermination	84.93±6.47^1,2^	81.23±4.72	86.96±3.53^1,2^	79.94±3.91

1 P<0.05, compared to group IV.

2 P<0.05, compared to group II.

### Pulmonary Artery Perfusion with TNF-α Ab Improved Oxygenation Index and Reduced CPB-induced Inflammation Significantly

The oxygenation index PaO_2_/FiO_2_ was not significantly different in the four groups of rabbits before CPB (P>0.05, Table 2). At CPB termination, the rabbits undergoing CPB had significantly poor oxygenation index compared to the rabbits without CPB (group I, II, and III vs. group IV, P<0.05, Table 2). However, pulmonary artery perfusion with TNF-α Ab during CPB improved the oxygenation index substantially (group II vs. group I and III, P<0.05, Table 2).

**Table 2:** Oxygenation index (PaO_2_/FiO_2_, mean ± SD, n = 10).

Group	I (CPB+LPD)	II (CPB+TNF-α Ab)	III (CPB only)	IV (No CPB)
Before CPB	551.40±23.32	538.90±18.45	560.40±31.32	553.60±25.79
CPB termination	421.40±35.40^1,2^	454.10±27.82^1^	387.80±36.71^1,2^	545.50±22.77^2^

1 P<0.05, compared to group IV.

2 P<0.05, compared to group II.

We then measured the parameters indicating pulmonary inflammation including neutrophil count and the levels of TNF-α and MDA in rabbit blood. We found that CPB significantly increased the neutrophil count of both left and right atrial blood (group I, II, and III vs. group IV, P<0.05, Table 3). The neutrophil count of left atrial blood was particularly higher than that of right atrial blood in rabbits undergoing CPB without the perfusion with TNF-α Ab (group I and III, P<0.05, Table 3), indicating a specific increase of pulmonary inflammation during CPB. Interestingly, compared to the rabbit undergoing CPB without TNF-α Ab, pulmonary artery perfusion with TNF-α Ab significantly decreased the neutrophil count of left atrial blood but without affecting the neutrophil level of right atrial blood (group II vs. group I and III, P<0.05, Table 3), indicating that the perfusion with TNF-α Ab might specifically reduce CPB-induced pulmonary inflammation. The inhibitory effect of TNF-α Ab on neutrophil accumulation lasted till CPB termination (Table 3). The effects of CPB and the perfusion with TNF-α Ab on the levels of TNF-α (Table 4) and MDA (Table 5) showed similar pattern as that on the neutrophil accumulation. CPB significantly increased TNF-α and MDA levels compared to the rabbits without CPB (group I, II, and III vs. group IV, P<0.05, Table 4 & 5) and particularly elevated TNF-α and MDA levels of left atrial blood to higher degree in rabbits undergoing CPB without the perfusion with TNF-α Ab (group I and III, P<0.05, Table 4 & 5). Pulmonary artery perfusion with TNF-α Ab significantly reduced TNF-α and MDA levels of left atrial blood (group II vs. Group I and III, P<0.05, Table 4 & 5) during CPB. These results suggest that pulmonary artery perfusion with TNF-α Ab could attenuate CPB-induced lung inflammation.

**Table 3:** Neutrophil count in blood samples (mean ± SD×10^9^/L, n = 10).

	Before CPB		5 min after releasing aortic clamp		CPB termination	
Group	Right atrium	Left atrium	Right atrium	Left atrium	Right atrium	Left atrium
I	1.89±0.16	1.83±0.14	4.66±0.63^1^	6.28±0.54^2,3,4^	3.98±0.63^1^	6.02±0.54^2,3^
II	1.72±0.19	1.80±0.15	3.94±0.40	4.70±1.37^2,4^	3.73±0.41	3.96±0.45^2,4^
III	1.81±0.11	1.89±0.17	4.97±0.48^1^	7.93±0.66^2,3^	4.12±0.41^1^	6.13±0.59^2,3^
IV	1.81±0.13	1.81±0.17	1.87±0.85	1.86±0.16^3,4^	1.89±0.11	1.88±0.17^3,4^

Group I: CPB+LPD. Group II: CPB+TNF-α Ab. Group III: CPB only. Group IV: No CPB.

1 P<0.05, compared to left atrium.

2 P<0.05, compared to group IV.

3 P<0.05, compared to group II.

4 P<0.05, compared to group III.

**Table 4:** TNF-α level in blood samples (mean ± SD, pg/ml, n = 10).

	Before CPB		5 min after releasing aortic clamp		CPB termination	
Group	Right atrium	Left atrium	Right atrium	Left atrium	Right atrium	Left atrium
I	93.95±7.66	95.08±13.44	164.04±13.13^1^	220.43±16.44^2,3,4^	158.09±8.34^1^	200.93±10.92^2,3^
II	96.22±8.24	93.47±4.74	162.61±5.26	185.27±11.78^2,4^	154.09±11.42	160.96±14.78^2,4^
III	95.01±7.29	94.72±7.40	203.24±18.43^1^	249.99±14.09^2,3^	190.46±10.66^1^	206.02±11.34^2,3^
IV	95.57±8.82	95.13±11.58	95.10±11.67	95.19±12.93^3,4^	95.64±6.13	95.61±7.02^3,4^

Group I: CPB+LPD. Group II: CPB+TNF-α Ab. Group III: CPB only. Group IV: No CPB.

1 P<0.05, compared to left atrium.

2 P<0.05, compared to group IV.

3 P<0.05, compared to group II.

4 P<0.05, compared to group III.

**Table 5:** MDA level in blood samples (mean ± SD, nmol/L).

	Before CPB		5 min after releasing aortic clamp		CPB termination	
Group	Right atrium	Left atrium	Right atrium	Left atrium	Right atrium	Left atrium
I	1.266±0.048	1.224±0.097	2.040±0.309^1^	2.546±0.462^2,3,4^	1.991±0.208^1^	2.496±0.376^2,3^
II	1.247±0.164	1.218±0.142	2.011±0.251	2.189±0.177^2,4^	1.894±0.211	2.016±0.178^2,4^
III	1.232±0.177	1.232±0.157	2.210±0.481^1^	2.816±0.648^2,3^	2.016±0.130^1^	2.505±0.419^2,3^
IV	1.228±0.057	1.230±0.083	1.224±0.137	1.215±0.103^3,4^	1.249±0.269	1.229±0.140^3,4^

Group I: CPB+LPD. Group II: CPB+TNF-α Ab. Group III: CPB only. Group IV: No CPB.

1 P<0.05, compared to left atrium.

2 P<0.05, compared to group IV.

3 P<0.05, compared to group II.

4 P<0.05, compared to group III.

### Pulmonary Artery Perfusion with TNF-α Ab Reduced CPB-induced TNF-α Over-expression and Apoptosis in Lung Tissue

**Figure 4:**
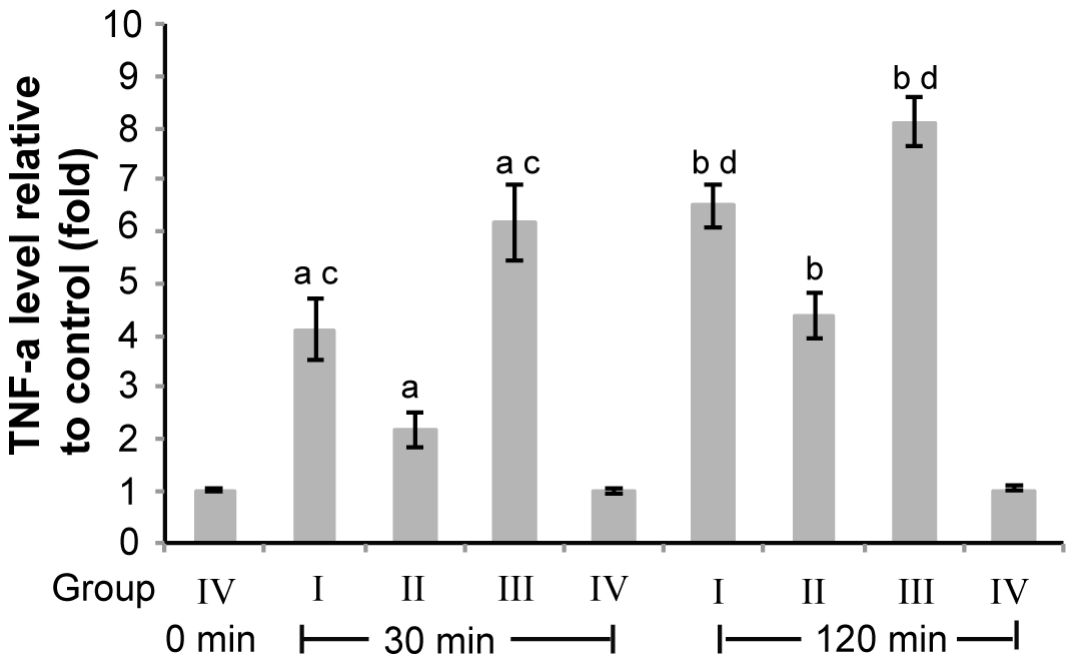
Pulmonary artery perfusion with TNF-α Ab reduced CPB-induced TNF-α over-expression in lung tissue. Group I: CPB+LPD; Group II: CPB+TNF-α Ab; Group III: CPB only; Group IV: Open chest no CPB. Lung tissues were collected after CPB lasted for 30 min and 120 min for group I–III, or when chest was just open, after chest was left open for 30 min and 120 min for group IV. Total RNA was extracted by the Trizol method. Quantitative real time RT-PCR was performed according to the description in the Methods. β-actin was used as the reference gene. TNF-α level was first normalized to the reference gene in each sample. The TNF-α level relative to that of the rabbits of group IV when the chest was just open was calculated used the equation 2^ΔΔCT^. n = 10. Data were presented as mean ± SD. ^a^P<0.05 group I, II, and III vs. group IV after CPB or chest open for 30 min. ^b^P<0.05 group I, II, and III vs. group IV after CPB or chest open for 120 min. ^c^P<0.05 group I and III vs. group II after CPB or chest open for 30 min. ^d^P<0.05 group I and III vs. group II after CPB or chest open for 120 min.

To further investigate the molecular mechanism underlying the protective effects of TNF-α Ab on lung during CPB, we then examined TNF-α expression in lung tissue by quantitative RT-PCR. Our result showed that TNF-α mRNA level was markedly induced by CPB (group I, II, and III vs. group IV, P<0.05, Figure 4), and the induction reached the highest at CPB termination. Pulmonary artery perfusion with TNF-α significantly reduced the CPB-induced up-regulation of TNF-α expression in lung tissue (group II vs. group I and III, P<0.05, Figure 4).

**Figure 5:**
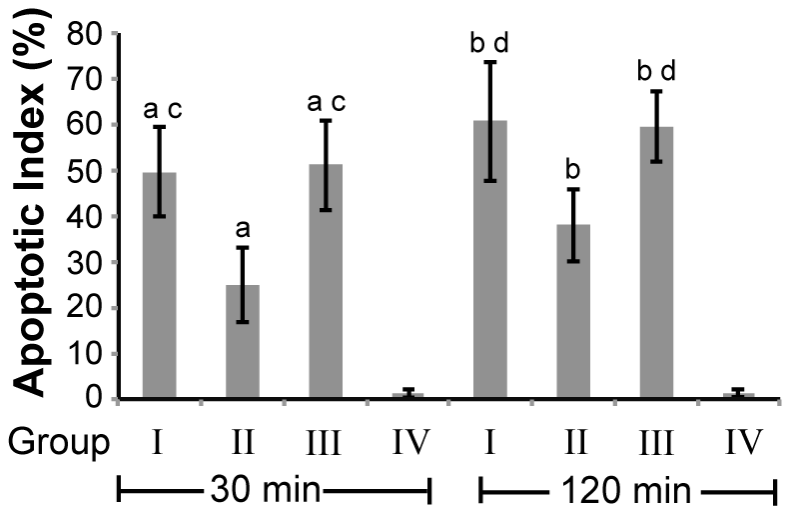
Pulmonary artery perfusion with TNF-α Ab decreased CPB-induced apoptosis in lung tissue. Group I: CPB+LPD; Group II: CPB+TNF-α Ab; Group III: CPB only; Group IV: Open chest no CPB. AI was determined according to the description in the Methods. Lung tissues were collected after CPB lasted for 30 min and 120 min for group I–III, or after chest was left open for 30 min and 120 min for group IV. ^a^P<0.05 group I, II, and III vs. group IV after CPB or chest open for 30 min. ^b^P<0.05 group I, II, and III vs. group IV after CPB or chest open for 120 min. ^c^P<0.05 group I and III vs. group II after CPB or chest open for 30 min. ^d^P<0.05 group I and III vs. group II after CPB or chest open for 120 min. n = 10. Data were presented as mean ± SD.

**Figure 6:**
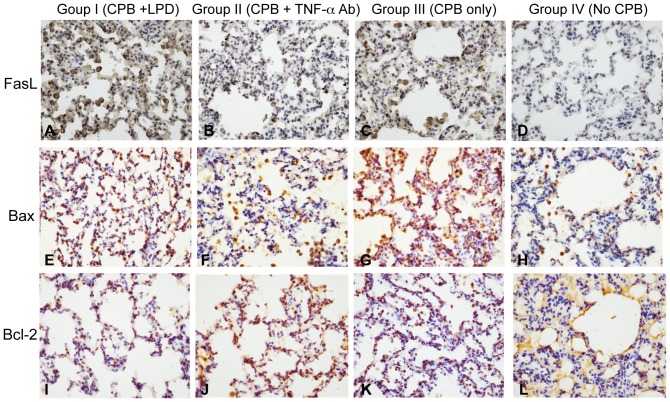
Pulmonary artery perfusion with TNF-α Ab reduced CPB-induced Bax and Fas over-expression and attenuated CPB-induced Bcl-2 down-regulation in lung tissues. Group I: CPB+LPD; Group II: CPB+TNF-α Ab; Group III: CPB only; Group IV: Open chest no CPB. Lung tissue sections of rabbits from group I, II and III at the CPB termination and from group IV when chest was left open for 120 min were stained with anti rabbit FasL (**A**–**D**), anti rabbit Bax (**E**–**H**), and anti rabbit Bcl-2 (**I–L**), respectively. Group I: A, E, and I; group II: B, F, and J; group III: C, G, and K; group IV: D, H, and L. The images (400x) were collected under microscope. The displayed images were the representative images.

Reduction of TNF-α expression and inflammation might be associated with a decrease of apoptosis in lung. Thus, we analyzed the apoptosis in lung tissue. Our TUNEL staining results showed that apoptosis was barely seen in the lung tissues of rabbits without CPB and was not affected by open chest procedure (group IV, Figure 5), but the apoptotic index (AI) increased significantly in lung tissues after CPB continued for 30 min and rose continuously until the termination of CPB (group I, II, III vs. group IV, P<0.05, Figure 5). Pulmonary artery perfusion with TNF-α Ab reduced the CPB-mediated AI induction in lung tissue considerably (Group II vs. Group I & III, P<0.05, Figure 5). This result suggests that the expression of proteins involved in apoptosis pathways including Bax, Bcl-2, and Fas ligand (FasL), might also change accordingly. Our immunohistochemical staining showed that the expression of FasL and Bax in alveolar epithelial cells of the rabbits undergoing CPB for 30 min were significantly increased (group I, II, and III vs. group IV, P<0.05, Table 6) and Bcl-2 level was decreased compared to those of the rabbits without CPB (group I, II, and III vs. group IV, P<0.05, Table 6). At CPB termination, the levels of FasL (Figure 6A–D) and Bax (Figure 6E–H) were further increased and Bcl-2 level (Figure 6I–L) was further reduced (Table 6, Figure 6). In contrast, the rabbits that underwent CPB and received pulmonary artery perfusion with TNF-α Ab exhibited significantly less FasL and Bax expression and higher Bcl-2 expression compared to the rabbits that had CPB without TNF-α Ab (Group II vs. Group I & III, P<0.05, Table 6, Figure 6). These results indicate that pulmonary artery perfusion with TNF-α Ab during CPB could reduce the CPB-induced apoptosis in lung tissue.

**Table 6:** Comparison of the protein levels of Bcl-2 (%), Bax (%), and FasL (%), and the ratio of Bcl-2/Bax in alveolar epithelial cells in different experimental groups (mean ±SD, n = 10).

	CPB for 30 min				CPB termination			
	I (CPB+LPD)	II (CPB+TNF-α Ab)	III (CPN only)	IV (No CPB)Chest openfor 30 min	I (CPB+LPD)	II (CPB+TNF-α Ab)	III (CPB only)	IV (No CPB)Chest openfor 120 min
FasL	41.24±5.63^1,2^	29.75±3.98^1^	53.84±8.64^1,2^	4.25±1.41	62.76±7.53^1,2^	31.78±5.43^1^	81.88±9.67^1,2^	4.32±1.32
Bax	35.74±7.34^1,2^	25.17±4.62^1^	56.78±9.12^1,2^	6.69±1.00	51.47±8.36^1,2^	42.25±6.46^1^	77.28±8.48^1,2^	6.96±1.03
Bcl-2	18.42±2.46^1,2^	21.13±1.47	15.25±1.98^1,2^	22.87±3.15	14.52±1.68^1,2^	17.36±2.01^1^	10.35±1.67^1,2^	23.54±2.34
Bcl-2/Bax	0.50±0.18^1,2^	0.84±0.27^1^	0.26±0.08^1,2^	3.13±0.49	0.28±0.09^1,2^	0.42±0.10^1^	0.14±0.07^1,2^	3.21±0.75

1 P<0.05, compared to group IV.

2 P<0.05, compared to group II.

## Discussion

CPB is known to induce a systemic inflammatory response that can increase the production of oxygen free radicals (OFR) and stimulate apoptosis, which consequently leads to lung damage [7]–[9]. Our results from TUNEL staining and EM show that CPB can induce apoptosis in type II alveolar epithelial and lung endothelial cells, which are the key cell types for maintaining normal lung function. TNF-α is thought to play a key role in mediating the CPB-induced lung damage and apoptosis in lung tissue [10], [11]. In this study, we found that inhibition of TNF-α activity by neutralizing antibody reduced CPB-induced apoptosis and inflammatory lung injury.

Our previous study shows that endotracheal intubation of TNF-α Ab does not suppress TNF-α expression in the lungs [4]. In contrast, in this study, we found that pulmonary artery perfusion of TNF-α Ab significantly reduced the mRNA level of TNF-α in the lungs of rabbits undergoing CPB. This result indicates that in addition to inhibiting the biological function of TNF-α, TNF-α Ab delivered by pulmonary artery perfusion could trigger the down-regulation of TNF-α expression. Lisby et al. demonstrated that TNF-α regulates its own mRNA synthesis in an autocrine manner in keratinocytes [12]. Thus, functional blockage of TNF-α could interrupt the autocrine TNF-α production. In fact, Szlosarek et al. have reported that the TNF-α neutralizing antibody infliximab reduces TNF-α mRNA level in ovarian cancer cells by blocking the autocrine production loop [13], which is consistent with our findings. Therefore, the inhibitory effect of TNF-α Ab on TNF-α expression seems to support that pulmonary artery perfusion of TNF-α Ab produces superior efficacy than endotracheal intubation. It has been shown that reduction of plasma TNF-α level can decrease the expression of adhesion molecules on neutrophil and endothelial cells so to reduce neutrophil accumulation in blood vessels, which subsequently attenuates neutrophil-mediated blood vessel leakage and reduces the neutrophil oxidative burst-induced damage of blood vessels and lung tissue [14].

Compared to endotracheal intubation, pulmonary artery perfusion not only allows more uniform and efficient delivery of TNF-α Ab to lungs, but also presents unique advantages. We used LPD, which is an extracellular preservation solution (EPS) containing low concentration of potassium, as the perfusion solution to deliver TNF-α Ab to lungs. It has been shown that EPS can preserve pulmonary function better than intracellular preservation solution by reducing pulmonary OFR production and attenuating OFR-induced lung damage in a rabbit model of pulmonary ischemia-reperfusion [15], [16]. In addition, pulmonary perfusion with EPS can wash the lung vessels to remove the residual blood cells that are responsible for initiating CPB-induced inflammatory reactions. Furthermore, the electrolyte composition, osmotic pressure, and osmotic pressure of LPD can prevent the activation of neutrophils during ischemia-reperfusion [17]. In fact, our results show that perfusion with LPD only in the absence of TNF-α Ab slightly improved oxygenation index and reduced CPB-induced pulmonary inflammation and apoptosis in rabbits undergoing CPB, however, the effects were less significant than those from the perfusion with TNF-α Ab.

In addition, because CPB interrupts pulmonary circulation, bronchial artery becomes the only vessel to supply blood to lung, leading to high metabolism and hyperthermia in lung, which consequently induces inflammatory reactions and causes ischemia-reperfusion injury in lungs. Thus, we used hypothermic LPD solution for pulmonary artery perfusion in this study to reduce those CPB-related adverse effects on lung. Numerous studies have demonstrated that hypothermic pulmonary perfusion can effectively reduce pulmonary metabolic rate, decrease OFR production, and increase plasma colloid osmotic pressure to prevent pulmonary edema [14], [18], [19]. Our results show that pulmonary artery perfusion with TNF-α Ab in hypothermic LPD significantly reduces CPB-induced apoptosis and prevents pulmonary edema.

Apoptosis is regulated by two evolutionary conserved signaling transduction pathways, the death receptor pathway and the mitochondrial pathway [20]. Fas/FasL system plays a key role in the death receptor pathway and interacts with pro-inflammatory reaction [21]. Bcl-2 and Bax plays an inhibitory and stimulatory role in the mitochondrial pathway, respectively. Delmotte et al. have demonstrated that TNF-α can stimulate the mitochondrial pathway to induce apoptosis [22]. Our results suggest that both apoptosis pathways are stimulated by CPB and pulmonary artery perfusion with TNF-α Ab attenuates CPB-induced apoptosis by blocking both apoptosis pathways.

In summary, our study suggests that pulmonary artery perfusion with TNF-α Ab during CPB can reduce CPB-induced inflammatory lung injury and improve lung function by substantially reducing pulmonary inflammation and apoptosis in lung. Our findings might shed new lights on new strategies for attenuating CPB-induced lung injury.
